# Podosomes, But Not the Maturation Status, Determine the Protease-Dependent 3D Migration in Human Dendritic Cells

**DOI:** 10.3389/fimmu.2018.00846

**Published:** 2018-04-30

**Authors:** Céline Cougoule, Claire Lastrucci, Romain Guiet, Rémi Mascarau, Etienne Meunier, Geanncarlo Lugo-Villarino, Olivier Neyrolles, Renaud Poincloux, Isabelle Maridonneau-Parini

**Affiliations:** Institut de Pharmacologie et de Biologie Structurale, IPBS, Université de Toulouse, CNRS, UPS, Toulouse, France

**Keywords:** dendritic cells, podosomes, 3D migration, toll-like receptor, maturation

## Abstract

Dendritic cells (DC) are professional Antigen-Presenting Cells scattered throughout antigen-exposed tissues and draining lymph nodes, and survey the body for pathogens. Their ability to migrate through tissues, a 3D environment, is essential for an effective immune response. Upon infection, recognition of Pathogen-Associated Molecular Patterns (PAMP) by Toll-like receptors (TLR) triggers DC maturation. Mature DC (mDC) essentially use the protease-independent, ROCK-dependent amoeboid mode *in vivo*, or in collagen matrices *in vitro*. However, the mechanisms of 3D migration used by human immature DC (iDC) are still poorly characterized. Here, we reveal that human monocyte-derived DC are able to use two migration modes in 3D. In porous matrices of fibrillar collagen I, iDC adopted the amoeboid migration mode. In dense matrices of gelled collagen I or Matrigel, iDC used the protease-dependent, ROCK-independent mesenchymal migration mode. Upon TLR4 activation by LPS, mDC-LPS lose the capacity to form podosomes and degrade the matrix along with impaired mesenchymal migration. TLR2 activation by Pam_3_CSK_4_ resulted in DC maturation, podosome maintenance, and efficient mesenchymal migration. Under all these conditions, when DC used the mesenchymal mode in dense matrices, they formed 3D podosomes at the tip of cell protrusions. Using PGE_2_, known to disrupt podosomes in DC, we observed that the cells remained in an immature status and the mesenchymal migration mode was abolished. We also observed that, while CCL5 (attractant of iDC) enhanced both amoeboid and mesenchymal migration of iDC, CCL19 and CCL21 (attractants of mDC) only enhanced mDC-LPS amoeboid migration without triggering mesenchymal migration. Finally, we examined the migration of iDC in tumor cell spheroids, a tissue-like 3D environment. We observed that iDC infiltrated spheroids of tumor cells using both migration modes. Altogether, these results demonstrate that human DC adopt the mesenchymal mode to migrate in 3D dense environments, which relies on their capacity to form podosomes independent of their maturation status, paving the way of further investigations on *in vivo* DC migration in dense tissues and its regulation during infections.

## Introduction

Dendritic cells (DC) are professional phagocytic and antigen-presenting cells, which populate the skin, mucosal surfaces, and most organs of the body (1, 2). They scan their environment in search for antigens, collect antigenic materials, and transport them via lymphatic vessels to draining lymph nodes where they trigger T lymphocyte activation and the onset of the adaptive immune response (2). Yet, while DC functions rely on their ability to migrate in tissues, the mechanisms underlying DC migration in three-dimensional (3D) environments are not completely understood.

Immature DC (iDC) have been shown *in vivo* to patrol randomly in tissues, such as the gut and the skin, constantly sampling the interstitium for potential pathogen entry (3, 4). Using micro-channels as an *in vitro* model of constrained migration, iDC alternate phases of rapid migration with phases of arrest, corresponding to random scanning of the environment and antigen capture (5–7).

During infection, Toll-like receptors (TLRs) mediate cellular responses to a large variety of pathogens (viruses, bacteria, and parasites) by inducing DC activation and maturation. DC maturation is characterized by changes in the surface expression pattern of CC-chemokine receptors. A decrease in the expression of CCR5, which is highly abundant in iDC and involved in their recruitment to the site of inflammation, is accompanied by an increase in the expression of CCR7 that is required for mature DC (mDC) migration toward its ligands CCL19 and CCL21 expressed by lymphatic vessels (2, 8–13). mDC also upregulate protein surface expression of antigen-presenting and co-stimulatory molecules for a proper activation of the T cell responses. Regarding the mechanisms of mDC migration, data from *in vivo* approaches and *in vitro* 3D collagen models showed that the so-called “amoeboid” migration mode, which refers to crawling amoeba, are used in porous environments. The amoeboid mode is integrin and protease independent, it involves cell contractility induced by activation of RhoA, the Rho-associated protein kinase ROCK and myosin II, and it is characterized by a round cell shape (1, 14–21).

Podosomes are adhesion cell structures, which are formed constitutively by macrophages, DC, and osteoclasts (22). The known podosome functions are cell adhesion, substrate rigidity sensing, and matrix degradation (22–28). In addition, podosomes and their cancer cell counterpart, invadopodia, are involved in the protease-dependent cell migration that takes place in dense 3D-environments. This mode is integrin-dependent and ROCK-independent. It is characterized by an elongated and protrusive cell shape, and it involves proteolytic degradation of the extracellular matrix (ECM) mediated by podosomes in macrophages and osteoclasts precursors (29–31). This migration mode is called mesenchymal migration. Interestingly, while TLR4-mediated human DC maturation by LPS induces the loss of podosomes (32–34), the TLR2-mediated maturation by Pam_3_CSK_4_ maintains podosome formation and stability (34), suggesting that DC migration capacity may be differentially regulated by TLR activation.

Therefore, in the present study, we hypothesized that the migration capacity of DC in 3D environments could be influenced by the architecture of the matrix, the cell maturation status, and the presence/absence of podosomes. We report that human monocyte-derived DC display amoeboid 3D migration in porous matrices of fibrillar collagen I, independent of their maturation status. We demonstrate that both iDC and mDC can adopt the mesenchymal migration mode to infiltrate 3D dense environments, a process that relies on their capacity to form podosomes.

## Materials and Methods

### Dendritic Cell Differentiation and Activation

Human monocytes were obtained from blood donors (Etablissement Français de Sang, EFS, Toulouse). For this report, written informed consents were obtained from all the donors under EFS contract n°21/PLER/TOU/IPBS01/2013-0042. According to articles L1243-4 and R1243-61 of the French Public Health Code, the contract was approved by the French Ministry of Science and Technology (agreement number AC 2009-921). All subjects gave written informed consent in accordance with the Declaration of Helsinki. Monocyte-derived macrophages and DC were differentiated as previously described (29, 35). Briefly, purified CD14^+^ monocytes were seeded in 24-well plates (5 × 10^5^ cells/well) with RPMI 1640 supplemented with 10% FCS, human IL-4 (Miltenyi Biotec) at 20 ng/mL, and human GM-CSF (Miltenyi Biotec) at 10 ng/mL. Cells were allowed to differentiate for 5–7 days. Fresh culture medium was added at day 3 of differentiation. For DC activation, cells were stimulated overnight with either LPS (from *Escherichia coli* O111:B4, Sigma-Aldrich) at 10 ng/mL, Pam_3_CSK_4_ (Synthetic triacylated lipoprotein, Invivogen) at 100 ng/mL, or PGE_2_ (Prostaglandin E2, kindly provided by Agnès Coste (PharmaDev, Toulouse)) at 5 μM, then harvested and used for the following assays. We also used ultra-pure LPS (from *Escherichia coli* O111:B4, Invivogen) and obtained similar results as those obtained with LPS from Sigma.

### Flow Cytometry

Immature DC, mDC-LPS, mDC-Pam_3_CSK_4_ and, iDC treated with PGE_2_ were harvested by gentle flushing with 1 mL of culture medium, centrifuged for 5 min at 340 g, incubated in staining buffer (PBS, 2 mM EDTA, 0.5% FBS) with a 1:100 dilution of Human TruStain FcX (Biolegend) for 5 min at room temperature. Cells were then stained in cold staining buffer for 25 min with fluorophore-conjugated antibodies (APC-Cy7 labeled anti-HLA-DR (clone: L243, 1/400), PE labeled anti-CD80 (clone: 2D10, 1/200), PerCP-Cy5.5 labeled anti-CD86 (clone: IT2.2, 1/400), Pacific Blue labeled anti-PD-L1 (clone: 29E.2A3, 1/400), PerCP-Cy5.5 labeled anti-CCR5 (clone: J418J1, 1/400), and PE labeled anti-CCR7 (clone: G043H7, 1/400)) from Biolegend. After staining, the cells were washed with cold staining buffer, centrifuged twice for 5 min at 340 g at 4°C, and analyzed by flow cytometry using LSR-II flow cytometer (BD Biosciences) and the associated BD FACSDiva software. Data were then analyzed using the FlowJo 7.6.5 software (TreeStar).

### 3D Migration Assay

Fibrillar (2.15 or 4 mg/mL) and gelled (5.15 mg/mL) collagen I, and Matrigel were prepared as previously described (29). Matrices (100 μL) were polymerized for 1 h and 30 min, respectively, in Transwell Invasion Chambers (BD Falcon) within 24-well companion plates. The viscoelasticity parameters of these matrices have been measured previously (29, 36). Matrigel, gelled collagen I at 5.15 mg/mL and fibrillar collagen I at 4 mg/mL display comparable viscoelasticity, while fibrillar collagen I at 2.15 mg/mL displays lower viscoelasticity. iDC, mDC-LPS, mDC-Pam_3_CSK_4_, and iDC treated with PGE_2_ (5 × 10^3^/transwell) were seeded on top of matrices. Migration experiments were conducted for 24–72 h, and the percentage of migrating cells, the distance of migration, and the number of membrane protrusions were quantified as described (29, 37). For live cell imaging of 3D migration (see Video S2 in Supplementary Material), pictures at the matrix surface and at 300 μm below the surface were recorded every 10 min during 13.5 h, using the 10× objective of an inverted video microscope (Leica DMIRB, Leica Microsystems, Deerfield, IL, USA) equipped with an incubator chamber to maintain constant temperature and CO_2_ levels. The chemokines CCL5, CCL19, and CCL21 (Immunotools) were added in the bottom chamber at 20 ng/mL. The mixture of protease inhibitors (PI mix) comprises E64c (100 μM), GM6001 (5 μM), aprotinin (0.04 TIU/mL), leupeptin (6 μM), and pepstatin (2 μM) (29). Y27632 was used at 20 μM. DMSO at the concentration of PI mix was used as a control in all experiments.

### Fluorescence Microscopy

Glass coverslips were coated with fibronectin (10 μg/mL, Sigma Aldrich) in PBS for 1 h at 37°C. Cells were seeded on fibronectin-coated coverslips (3 × 10^5^/well in 24-well plate), left to adhere for 1–3 h and stimulated as indicated. DC were then fixed and stained with anti-Vinculin (Sigma-Aldrich) and Texas-Red-coupled phalloidin as previously described (38) and imaged using the 60 × 1.4 objective on an FV1000 confocal microscope (Olympus). The number of cells displaying podosomes was counted using a Leica DM-RB fluorescence microscope on least 100 cells per experimental conditions.

### 3D Podosome Staining and Imaging

At the end of migration experiments, matrices were fixed with 3.7% (w/v) paraformaldehyde (PFA) and 15 mM sucrose for 45 min at room temperature. PFA was quenched with 50 mM NH_4_Cl for 5 min. Cells embedded in matrices were permeabilized with PBS–Triton X-100 0.1% supplemented with 3% (w/v) BSA to perform saturation at the same time for 1 h. Afterward, cells were stained anti-vinculin (Sigma-Aldrich) and secondary AlexaFluor 488-coupled secondary antibody, phalloidin-Texas Red (Invitrogen) and DAPI (0.5 mg/mL; Sigma-Aldrich). Cells were imaged using an Olympus/Andor CSU-X1 spinning disk with a 60× objective.

### Scanning Electron Microscopy

Scanning electron microscopy (SEM) observations were performed as previously described (39, 40). Briefly, at the end of the 3D migration assay, cells and matrices were fixed in 2.5% glutaraldehyde/3.7% PFA/0.1M sodium cacodylate (pH 7.4) and dehydrated in a series of increasing ethanol. Critical point was dried using carbon dioxide in a Leica EMCPD300. After coating with gold, cells were examined with an FEI Quanta FEG250 scanning electron microscope.

### Tumor Cells and Spheroid Culture

Spheroids were generated as previously described (36). Briefly, 24-well tissue culture plates were coated with 500 μL of 2% agar per well. The human breast tumor cell line SUM159PT (10^3^ cells/20 μL) was plated in the lid of tissue culture plates. After 7 days, each spheroid was transferred into wells with 500 μL culture medium. Preliminary studies have established that after 20–24 days of culture, spheroids reached a diameter of ~400 μm. DC staining was performed using the cell-live permeant probe CellTracker Red CMPTX (Molecular Probes, Invitrogen) at 0.5 μM in PBS, as described by the manufacturer. DC at day 7 of differentiation were distributed (10^4^ cells) into agar-coated wells containing a single spheroid and co-incubated for 3 days. Formalin-fixed spheroids stained with DAPI were imaged in chambers (CoverWell PCI-1.0; Grace Bio-Labs, Bend, OR) using a Zeiss LSM710 microscope (10× objective, NA 0.3, voxel size 1.3 μm × 1.3 μm × 5.5 μm) with a multiphoton source at 715 nm (coherent Chameleon) for z-stack acquisition of DAPI and CellTracker fluorescence. With the cell counter plugin of ImageJ software (National Institutes of Health, Bethesda, MD, USA), CellTracker-stained DC associated to spheroids were counted. DC were classified “out of spheroids” when located in the first line of nuclei, and “inside” when entering the first line of nuclei. At least three spheroids per condition were used.

### Statistics

A Wilcoxon matched-paired signed rank test was used for statistical analyses performed using GraphPad Prism 6.0 (GraphPad Software Inc.). The *P* < 0.05 was considered significant.

## Results

### Immature DC Adopt Either the Amoeboid or the Mesenchymal Migration Mode, and Form Podosomes Depending on the Matrix Architecture

**Figure 1:**
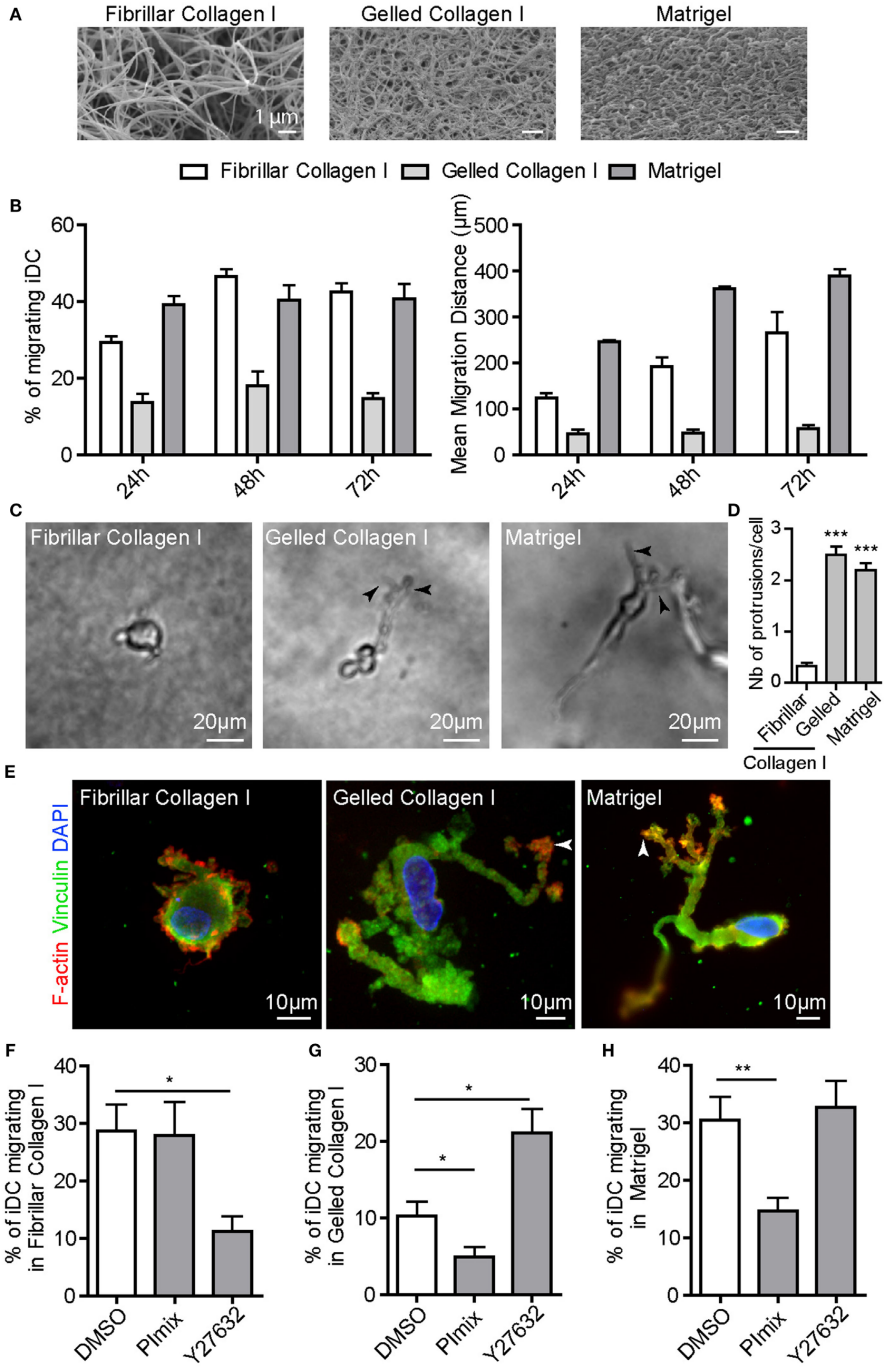
Immature DC (iDC) adopt either the amoeboid or the mesenchymal migration mode depending on the matrix architecture. iDC were seeded on top of a thick layer of fibrillar or gelled collagen I, or Matrigel polymerized in culture transwell inserts. **(A)** Scanning electron microscopy pictures revealing the porous (fibrillar collagen I) *versus* dense (gelled collagen I and Matrigel) architecture of the matrix. **(B)** The percentage of migrating cells and the mean migration distance of iDC in fibrillar, gelled collagen I or Matrigel were measured. Results are expressed as mean ± SEM of at least three independent experiments. **(C)** Bright field images of iDC within matrices were taken using an inverted video microscope and illustrate the round cell morphology in fibrillar collagen I and the elongated and protrusive cell morphology in gelled collagen I and Matrigel. **(D)** Quantification of the number of membrane protrusions per cells. Results are expressed as mean ± SEM of at least 100 cells counted per conditions. **(E)** After migration in matrices in Transwell inserts for 24 h, samples were fixed, permeabilized, and stained with anti-vinculin (green), phalloidin Texas-Red (red) and DAPI (blue). iDC form 3D podosomes, F-actin-, vinculin-enriched structures at the tip of membrane protrusions when migrating in dense matrices of gelled collagen I and Matrigel (arrowheads). **(F–H)**, The percentage of iDC migrating in fibrillar collagen I **(F)**, gelled collagen I **(G)** or Matrigel **(H)** was measured in control or drug-treated cells (PImix or Y27632). Results are expressed as mean ± SEM of at least three independent experiments.

To study the 3D migration ability of human monocyte-derived DC, we used different matrices presenting distinct architectures that were polymerized in transwells as thick layers (>1 mm) (29). Fibrillar collagen I is a porous matrix used to mimic classical stromal/interstitial ECM, and Matrigel is a dense matrix composed of a mixture of ECM proteins particularly rich in laminin and collagen IV (Figure 1A) (41). Since Matrigel and fibrillar collagen I have distinct biochemical compositions, we also used collagen I polymerized as a dense gel, called gelled collagen I, which displays a dense architecture and viscoelasticity parameters similar to Matrigel (Figure 1A). As shown in Figure 1B, iDC migrated in fibrillar collagen I, Matrigel and, to a lower extent, in gelled collagen I. iDC, imaged inside gelled collagen I and Matrigel, displayed an elongated cell shape with long protrusions (Videos S1 and S2 in Supplementary Material; Figure 1C). In fibrillar collagen I, iDC displayed a round cell shape (Video S1 in Supplementary Material; Figure 1C). The number of cells forming membrane protrusions and the number of membrane protrusions per cell were quantified. More than 90% of iDC migrating in gelled collagen I and Matrigel form membrane protrusions (mean of two to three protrusions per cell), while cells in fibrillar collagen I had occasional protrusions (Figure 1D).

Next, we examined whether iDC migrating in dense matrices form 3D podosomes, as these structures are instrumental for matrix remodeling and protease-dependent migration (26, 29, 30, 42). Cell staining in dense matrices revealed that the tips of membrane protrusions were enriched in F-actin and vinculin (Figure 1E), indicative of the presence of 3D podosomes. When iDC migrated inside fibrillar collagen I, they did not form 3D podosomes as observed with F-actin and vinculin at the subcortical area (Figure 1E).

To further characterize the migration mode of iDC, we used the ROCK inhibitor Y27632 or a mix of protease inhibitors [PImix (29)], which inhibit the amoeboid and mesenchymal migration modes, respectively. In the presence of Y27632, iDC migration in fibrillar collagen I was strongly inhibited, whereas it was not affected by PImix (Figure 1F). Since Matrigel and fibrillar collagen I have distinct viscoelasticity parameters, we also used fibrillar collagen I at 4 mg/mL, which displays similar viscoelasticity parameters as Matrigel but the same porous architecture as fibrillar collagen I at 2.15 mg/mL (29). Using this matrix, we showed that iDC migration was inhibited by Y27632 (43 + 1.2% *versus* 1.6 + 2.4%, *P* = 0,0022). On the contrary, while Y27632 did not affect cell migration in gelled collagen I and Matrigel, PImix significantly reduced it (Figures 1G,H). We verified that podosomes displayed a proteolytic activity on gelatin-FITC coated glass coverslips (Figures S1A–D in Supplementary Material) that was markedly reduced in the presence of PImix (Figures S1C,D in Supplementary Material). As previously reported (43), protease inhibitors induced podosome dissolution (Figures S1A,B in Supplementary Material). By contrast, Y27632 did not affect podosome formation and matrix degradation activity (Figures S1A–D in Supplementary Material).

Taking together the results on the cell shape, the effect of inhibitors and the formation of 3D podosomes, we conclude that iDC use the amoeboid migration mode in porous matrices of fibrillar collagen I and the mesenchymal migration mode in dense matrices of gelled collagen I and Matrigel. Thus, iDC adapt their migration mode to the architecture rather than the composition of the 3D matrix. As these results are comparable to those obtained with human monocyte-derived macrophages (26, 29), we decided to compare the 3D migration capacity of these two cell types that form podosomes [Figure S2A in Supplementary Material (23, 27, 29)]. Macrophages and iDC were differentiated from monocytes isolated from the same donors. The percentage of macrophages and iDC migrating in fibrillar collagen I was similar, but macrophages covered a longer distance than DC at 24 h (Figure S2B in Supplementary Material). In Matrigel, iDC displayed a higher capacity to infiltrate the matrix compared to macrophages (percentage of migrating cells and migration distance) during the first 24 h; however, both cell types migrated equally after 3 days (Figure S2C in Supplementary Material). Therefore, macrophages and iDC display similar ability to migrate in both types of matrix.

### Podosomes, Rather Than the Maturation Status, Determine the Ability of DC to Perform Mesenchymal 3D Migration

**Figure 2:**
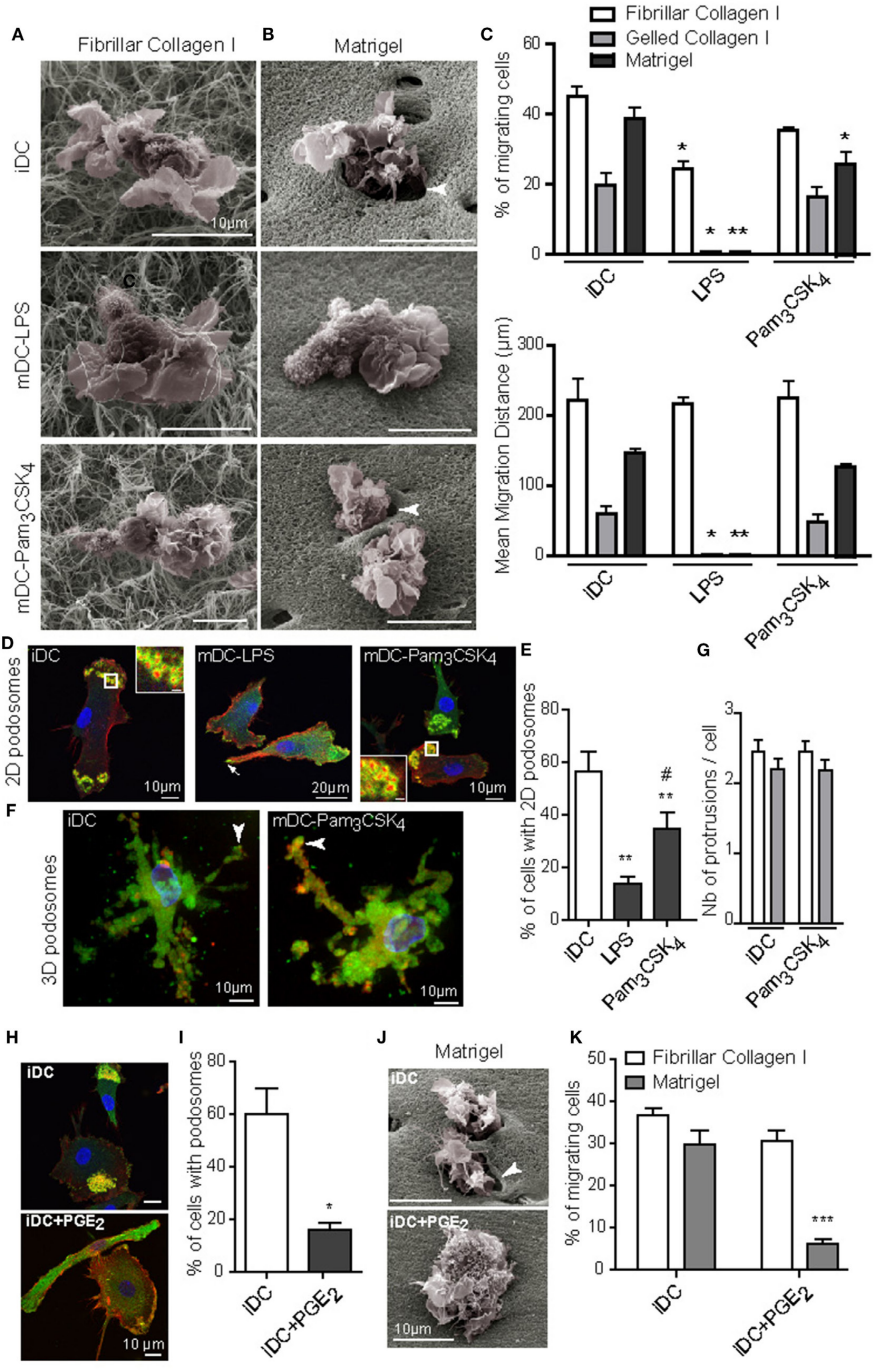
TLR4 and PGE_2_, but not TLR2, activation induced podosome dissolution and abolished 3D mesenchymal migration of dendritic cells (DC). Immature DC (iDC) were left untreated or stimulated with LPS (10 ng/mL) or Pam_3_CSK_4_ (100 ng/mL) for 16 h. **(A,B)**, Morphology of cells and interaction with the surrounding matrix of fibrillar collagen I **(A)** or Matrigel **(B)** were visualized by scanning electron microscopy. Note the presence of holes in the matrix around iDC and mature DC (mDC)-Pam_3_CSK_4_ penetrating Matrigel (arrowheads), which were not seen in mDC-LPS. **(C)** The percentage of migrating cells and the mean migration distance in fibrillar, gelled collagen I or Matrigel, were measured after 24 h. Results are expressed as mean ± SEM of at least three independent experiments. **(D)** 2D podosomes were stained with an anti-vinculin Ab (green), phalloidin Texas-Red to detect F-actin (red), and DAPI to stain nuclei (blue) (scale bar inset: 2 μm). **(E)** The percentage of cells forming 2D podosomes was quantified. Results are expressed as mean ± SEM of seven independent experiments. **(F)** 3D podosomes of iDC and mDC-Pam_3_CSK_4_ in gelled collagen I were stained with an anti-vinculin Ab (green), phalloidin Texas-Red to detect F-actin (red), and DAPI to stain nuclei (blue). **(G)** The number of membrane protrusions per cell was quantified in gelled collagen I (white) and Matrigel (gray). **(H–K)**, iDC were left untreated or treated with PGE_2_ (5 μM) for 16 h. **(H)** Podosomes were stained with an anti-vinculin Ab (green), phalloidin Texas-Red to detect F-actin (red), and DAPI to stain nuclei (blue). **(I)** the percentage of cells forming 2D podosomes was quantified. Results are expressed as mean ± SEM of seven independent experiments. **(J)** Morphology of cells and interaction with the surrounding matrix of Matrigel were visualized by scanning electron microscopy. Note the presence of holes in the matrix around iDC (arrowhead), which were not seen in iDC + PGE_2_. **(K)** The percentage of cells migrating in fibrillar collagen I or Matrigel was measured after 24 h. Results are expressed as mean ± SEM of at least three independent experiments. *P < 0.05; **P < 0.01; ***P < 0.001 compared to iDC condition. ^#^*P* < 0.05 compared to mDC-LPS condition.

Next, we investigated the influence of DC maturation on the 3D migration capacity. iDC were stimulated by LPS (TLR4 agonist) or Pam_3_CSK_4_ (TLR2 lipopeptide agonist) to generate mDC-LPS or mDC-Pam_3_CSK_4_. LPS- and Pam_3_CSK_4_-induced DC maturation was confirmed by the up-regulation of maturation markers at the cell surface, such as HLA-DR, CD80, CD86 and PD-L1 (Figures S3A,B in Supplementary Material). On top of porous fibrillar collagen I, both iDC, mDC-LPS and mDC-Pam_3_CSK_4_ exhibited a round cell shape with large membrane ruffles and blebs, as shown by SEM (Figure 2A). They efficiently infiltrated the matrix and covered a similar migration distance (Figure 2C). SEM pictures of Matrigel revealed that iDC and mDC-Pam_3_CSK_4_ remodeled the matrix by forming infiltrating holes, while mDC-LPS failed to do so (Figure 2B). In line with these observations, unlike mDC-LPS that did not infiltrate Matrigel or gelled collagen I, iDC and mDC-Pam_3_CSK_4_ infiltrated dense matrices and covered a similar migration distance (Figure 2C). Similar to iDC, mDC-Pam_3_CSK_4_ displayed a round cell shape in fibrillar collagen I and an elongated cell shape in dense matrices (Video S3 in Supplementary Material).

Of note, we observed that LPS treatment triggered DC maturation along with podosome dissolution (32–34). By contrast, podosomes were maintained when DC maturation was induced by Pam_3_CSK_4_ (34). These previous observations were confirmed in Figures 2D,E. iDC and mDC-Pam_3_CSK_4_ formed 3D podosomes in gelled collagen I (Figure 2F) and membrane protrusions in dense matrices (Figure 2G). Altogether, these results suggest that the ability of DC to migrate in dense matrices is independent of their maturation status, but it relies on the capacity to form podosomes.

To further characterize the role of podosomes in DC mesenchymal migration, we looked for a way to trigger podosome dissolution without inducing DC maturation. To address this question, PGE_2_ was used as a potent inducer of podosome dissolution (44), and thus we examined whether it modifies the DC maturation status. PGE_2_ did not induce DC maturation, as the cell surface expression of HLA-DR, CD80, and CD86 remained unchanged compared to iDC (Figure S3B in Supplementary Material), and we confirmed its capacity to disrupt podosomes (Figures 2H,I). When PGE_2_-treated iDC were seeded on top of matrices, we observed that both matrix proteolysis and migration in Matrigel were impaired without affecting amoeboid migration in fibrillar collagen I (Figures 2J,K).

Collectively, these results indicate that the presence of podosomes, rather than the maturation status of DC, determines the ability to migrate in dense 3D environments.

### CC-Chemokines Regulate 3D Migration of DC

**Figure 3:**
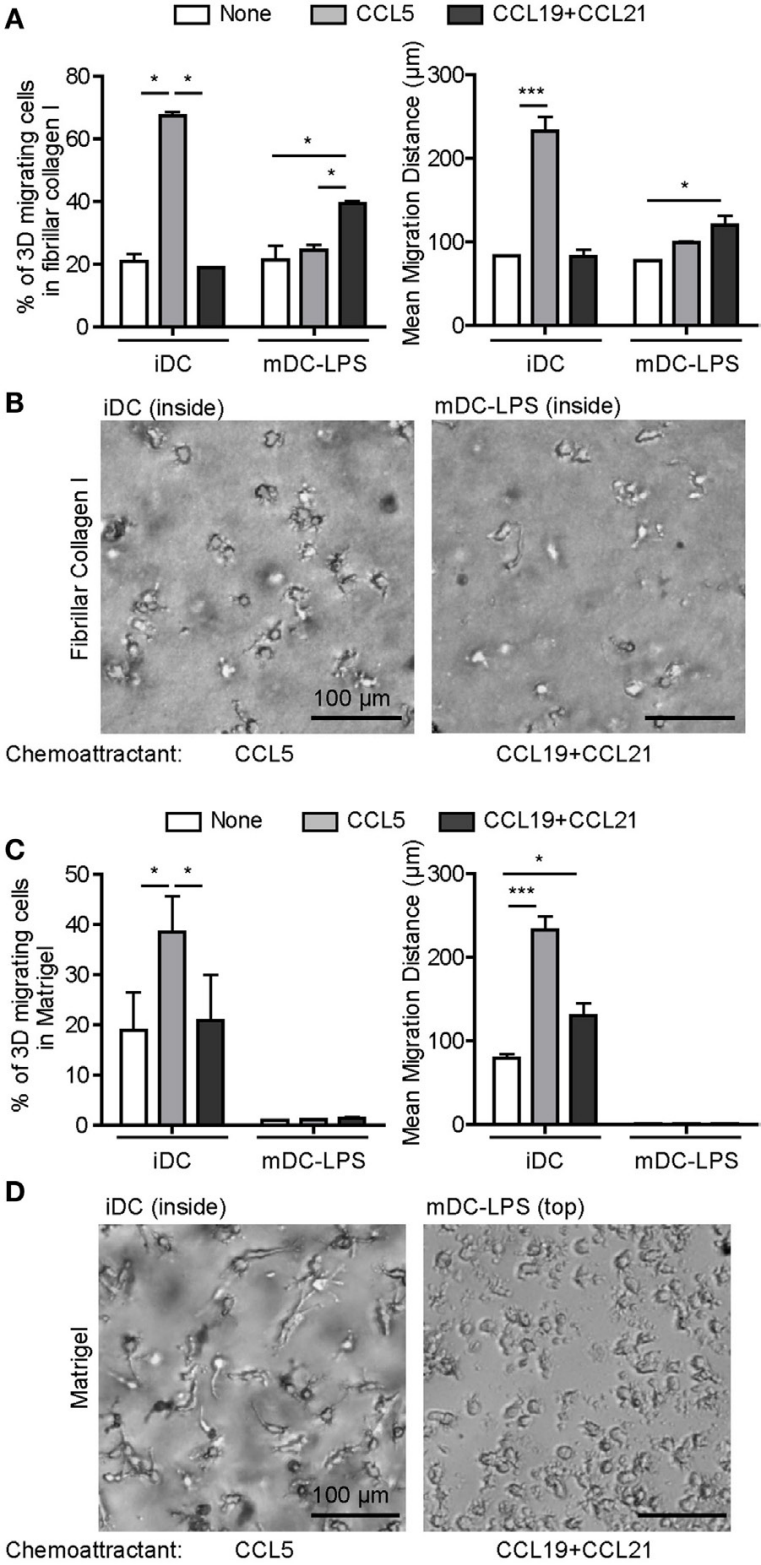
Chemokines regulate three-dimensional (3D) migration of dendritic cells (DC). **(A)** Immature DC (iDC) and mature DC (mDC)-LPS were seeded on top of a matrix of fibrillar collagen I and the percentage of migrating cells (left) or the mean migration distance (right) were monitored after 6 h, when none (white), CCL5 (gray), or a mixture of CCL19 and CCL21 (black) were added in the lower chamber as chemoattractant. Results are expressed as mean ± SEM of three independent experiments. *P < 0.05; ***P < 0.001 compared to iDC condition. **(B)** Bright field images of iDC and mDC-LPS within the matrix of fibrillar collagen I were taken using an inverted video microscope and illustrate the round cell morphology in response to CCL5 or a mixture of CCL19 and CCL21 chemokines, respectively. **(C)** iDC and mDC-LPS were seeded on top of Matrigel and the percentage of 3D migrating cells (left) or the mean migration distance (right) were monitored after 6 h, when none (white), CCL5 (gray), or a mix of CCL19 and CCL21 (black) were added in the lower chamber as chemoattractant. Results are expressed as mean ± SEM of three independent experiments. *P < 0.05; ***P < 0.001. **(D)** Bright field images of iDC within Matrigel were taken using an inverted video microscope and illustrate the elongated cell morphology in response to CCL5, while mDC-LPS remained on top of the matrix under the influence of CCL19 and CCL21 chemokines.

Since mDC-LPS migration in Matrigel is impaired, we next investigated whether it could be restored by chemokines. Upon TLR4 activation by LPS, the expression pattern of CC-chemokine receptors was modified with decreased expression of CCR5 and enhanced expression of CCR7 (Figure S4A in Supplementary Material), as previously described (8). Consequently, we used CCL5 or a combination of CCL19 and CCL21, as ligands for CCR5 and CCR7, respectively. In fibrillar collagen I, the percentage of migrating cells and the distance covered by iDC and mDC-LPS were enhanced by CCL5 and the combination of CCL19 and CCL21, respectively (Figure 3A). Under the influence of chemokines, both iDC and mDC-LPS exhibited the characteristic amoeboid round cell shape in fibrillar collagen I (Figure 3B), and their migration capacity was inhibited by Y27632 (Figures S4E,F in Supplementary Material). In Matrigel, however, CCL5 strongly increased the 3D migration and the distance covered by iDC, while CCL19 and CCL21 had no effect on these cells (Figure 3C). CCL5 also enhanced iDC migration in gelled collagen I (Figure S4B in Supplementary Material), and the percentage of migrating iDC under the influence of CCL5 was inhibited by PImix in Matrigel and gelled collagen I (Figures S4G–H in Supplementary Material). Importantly, none of these cytokines triggered mDC-LPS migration in Matrigel. Under the influence of CCL5, iDC displayed an elongated cell shape in Matrigel while mDC-LPS remained on top of the matrix (Figure 3D). Finally, we did not observe any influence of these chemokines on the capacity of iDC and mDC-LPS to degrade the matrix (Figures S4C,D in Supplementary Material).

Due to the switch in CC-chemokine receptor expression pattern, we observed that the amoeboid migration of iDC and mDC-LPS is, as expected, influenced by CCR5 and CCR7 ligands, respectively. While the mesenchymal migration of iDC is influenced by CCL5, CCL19 and CCL21 failed to trigger mDC-LPS infiltration in Matrigel. We infer that this is likely due to the dissolution of podosomes induced by LPS resulting in mDC-LPS inability to degrade the matrix.

### Immature DC Use Both Migration Modes in Tumor Cell Spheroids

**Figure 4:**
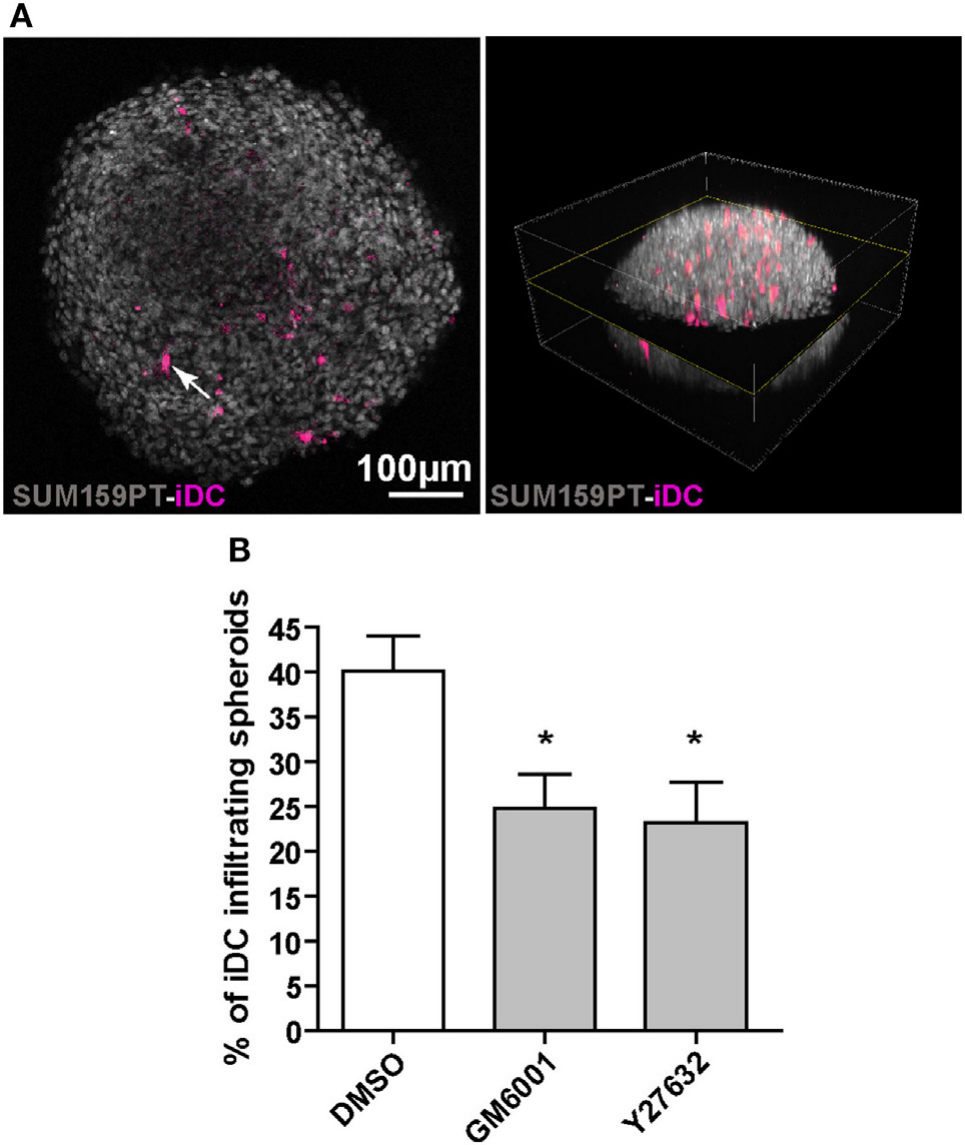
Immature dendritic cells (iDC) adopt both amoeboid and mesenchymal three-dimensional (3D) migration in tumor spheroids. SUM159PT cell spheroids were coincubated for 3 days with CellTracker-stained DC, with or without drugs. **(A)** Multiphoton acquisition of DAPI stained spheroids (gray) infiltrated by CellTracker-stained iDC (purple). A spheroid cross-section (left) set in the 3D spheroid reconstitution is shown (right). The arrow indicates a CellTracker-stained iDC located inside the spheroid. **(B)** Quantification of iDC infiltration into spheroids, with or without inhibitors. Results are expressed as the percentage of iDC inside spheroids (100% corresponds to iDC inside plus iDC at the periphery). Results are expressed as mean ± SEM of five independent experiments. *P < 0.05.

To examine the migration of iDC in a tissue-like 3D environment, we used spheroids of the breast carcinoma cell line SUM159PT, which secrete several ECM proteins including fibronectin, laminin, and collagen IV (36). DC were stained with CellTracker and co-cultured with spheroids in the presence of DMSO (vehicle), the pan-matrix metalloprotease inhibitor GM6001 (a component of PImix), or Y27632. After 3 days of co-culture, iDC infiltrated in spheroids were visualized using multiphoton microscopy (Figure 4A) and quantified. As shown in Figure 4B, iDC efficiently infiltrated tumor spheroids and both GM6001 and Y27632 significantly decreased the percentage of iDC inside spheroids (Figure 4B). Therefore, iDC are able to use both the mesenchymal and amoeboid modes in a complex tissue-like environment.

## Discussion

This study extends our knowledge on the migration ability of human DC in 3D environments and its modulation during TLR-induced DC maturation. We report the following novel findings: (1) both iDC and mDC use the amoeboid mode to migrate in porous 3D collagen I; (2) only DC forming podosomes migrate in dense environments, independent of their maturation status or the presence of chemokines; and (3) iDC use both the mesenchymal and amoeboid migration modes to infiltrate tumor cell spheroids.

Our first attempt was to investigate the migration capacity of iDC and the influence of TLR-induced maturation in 3D environments using different matrices with distinct architectures (29). We observed that, independent of their maturation status, DC adopt the amoeboid mode to migrate in porous collagen I as characterized by ROCK dependency and round cell shape. Hence, both iDC and mDC behave like other leukocytes, namely monocytes, T lymphocytes, macrophages, and neutrophils (14, 18, 29, 38, 45) with the 3D amoeboid mode as a common feature to migrate in porous 3D environments.

We demonstrate that, independent of their maturation status, DC migrate in dense matrices if they form podosomes as examined in 2D and 3D settings. Both the TLR4 and TLR2 agonists, LPS and Pam_3_CKS_4_, respectively, induce DC maturation. However, although LPS triggers podosome dissolution and loss of the mesenchymal migration capacity, Pam_3_CKS_4_ maintains podosomes and mesenchymal migration in dense matrices. These results suggest that in the context of infectious diseases, activation of distinct TLR triggers DC maturation with distinct impact on podosome formation and matrix degradation capacity. Using PGE_2_, which is synthesized downstream of LPS stimulation and mediates podosome dissolution (44, 46), we showed that it maintained DC in an immature state and abolished migration in Matrigel concomitantly to podosome disruption. In contrast to the TLR4 signaling pathway, PGE_2_ is not produced upon TLR2 stimulation (47), and thus podosomes are maintained. Due to the unique ability of DC to dissolve their podosomes, this study further supports the critical role of these cell structures in 3D migration in dense environments. We also report that when DC form podosomes in 2D, they form 3D podosomes during migration in dense 3D environments, as previously described in macrophages (29, 30, 36). Altogether, these data provide evidence that when DC form podosomes, they have the dual 3D migration ability, using the amoeboid mode in a porous matrix and the mesenchymal mode in a dense environment.

Among leukocytes, only macrophages and osteoclast precursors perform mesenchymal migration, which is functionally linked to their capacity to form podosomes (26, 29, 31, 37, 38, 42, 48). Macrophages that form podosomes (in 2D and 3D environments) are able to degrade, ingest, and compact the matrix to form tunnels and create paths allowing migration into dense matrix (26, 30). Interestingly, gene deletion or knockdown of podosome effectors such as Hck, WASp, or Filamin A translate in reduced podosome stability, ECM proteolysis and mesenchymal migration without any effect on amoeboid migration (39, 49, 50). Conversely, the HIV-1 protein Nef, which stabilizes podosomes and increases their proteolytic activity, enhances the mesenchymal migration of human macrophages (37). Thus, a pathogen able to modify podosome formation and function alters the 3D migration of its host cell in dense matrices (37). A few other studies described the influence of pathogens on podosomes and the consequences on DC migration mainly studied in 2D. In 2D, the influence of podosomes on cell migration is likely related to their adhesion property rather than to their proteolytic activity. Gram-positive *versus* Gram-negative bacteria have distinct effects on podosomes. Gram-negative bacteria, such as *Neisseria meningitidis or Salmonella enteritidis*, induce podosome dissolution in DC associated with enhanced migration on 2D surface in a LPS- and TLR4-dependent manner (34). Gram-positive bacteria, such as *Staphylococcus aureus* or *Streptococcus pneumoniae*, maintain podosomes and do not influence 2D migration of DC (34). Infection of human DC with the parasite *Toxoplasma gondii* induces a rapid dissolution of podosomes (51, 52) and enhanced amoeboid 3D migration in fibrillar collagen I (53). Finally, *Helicobacter pylori* is able to induce podosome formation in cells devoid of podosomes. Hepatocytes infected with *H. pylori* form podosomes, degrade the matrix, and exhibit diminished 2D cell migration (54). Altogether, these data show that several pathogens target podosomes with potential consequences on cell adhesion, matrix degradation, and cell migration, likely influencing tissue immunopathology and activation of the adaptive immune response.

Although podosomes in macrophages and DC are involved in mesenchymal migration, they exhibit distinct behaviors in response to protease inhibitors. The presence of protease inhibitors in the culture medium disrupted podosomes in DC, as previously observed with a cathepsin B inhibitor (43), but they exhibited no effect on podosomes in macrophages (29). Thus, proteases in macrophages and DC are involved in the proteoly-tic activity of podosomes toward the ECM and regulate podosome dynamics only in DC. Additional comparative experiments are required to further characterize specific properties of podosomes between macrophages and DC.

Chemokines, together with the switch in CC–chemokine receptor expression operating during DC maturation, tightly regulate DC migration (2). Here, we observed that chemokines enhanced the 3D migration capacities of DC and the migration distance in both dense and porous matrices, but none of them modulated their matrix degradation capacities. In addition, when DC are devoid of podosomes, chemokines are unable to trigger mesenchymal migration. Thus, chemokine receptors do not directly govern the 3D migration mode used by DC, but they facilitate their intrinsic migratory capacities. In response to CCL19, the small Rho-GTPase Cdc42 is essential for an efficient unipolar and directional migration of mDC-LPS both *in vivo* and *in vitro* in a 3D collagen matrix (16), likely explaining the increased distance covered by DC in matrices. Moreover, PGE_2_ induced during TLR4-mediated maturation of DC plays a critical role in CCR7 expression and signaling for an efficient migration of mDC-LPS toward CCL19 and CCL21 (55–57). Our results are in line with these previous studies since CCR7 expression is up-regulated and the amoeboid migration of mDC-LPS is enhanced in response to CCL19 and CCL21, probably facilitating the amoeboid migration to lymph nodes (1). The dual capacity of CCL5 to enhance both amoeboid and mesenchymal migration in iDC suggests that activation of CCR5 might support cell migration in all types of matrix architecture. Whether common or distinct molecular mechanisms are involved downstream of CCR5 activation to stimulate both migration modes remains to be determined.

The role of proteases in DC migration has been already reported showing that the Matrix Metallo-Proteases MT1-MMP and MMP9 regulate CCL5-induced iDC migration (58, 59). Interestingly, MT1-MMP localizes on podosome protrusions in iDC where it mediates ECM degradation (60–63). Moreover, MMP9 and MMP2 activities are also involved in Langerhans and dermal DC emigration from *ex vivo* murine and human epidermis (64). However, in these studies, it was not investigated whether cells form podosomes and exhibit the characteristics of mesenchymal motility. In tumor cell spheroids, we found that the pan-MMP inhibitor reduced cell infiltration, indicating that MMPs are involved in mesenchymal migration used by DC to infiltrate a tissue-like environment.

*In vivo*, existence of podosomes in myeloid cells and their role in 3D migration have not been formally demonstrated, but correlations have been provided between the capacity of cells to form podosomes in 2D and their migration capacity *in vivo*. In the past, we showed that deficiency of the tyrosine kinase Hck in macrophages reduces podosome stability and mesenchymal migration *in vitro*, which correlated with impaired migration of macrophages in the peritoneal cavity during inflammation (39, 49). Conversely, the HIV-1 protein Nef, which increases the podosome stability and mesenchymal migration *in vitro*, enhances the recruitment of macrophages in tumors in Nef-transgenic mice (37). Here, we showed that iDC infiltration in tumor cell spheroids was in part dependent on MMP activity, suggesting that DC might use proteases, probably through podosome formation, to migrate in tumors. Interestingly, abolition of tumor cell-secreted PGE_2_ enhanced conventional DC1 infiltration in tumors, and this was associated with tumor rejection (65). Although the impact of PGE_2_ on the actin cytoskeleton was not addressed, we hypothesize that tumor-derived PGE_2_, by partly disrupting podosomes, may prevent protease-dependent DC accumulation and immune-dependent tumor rejection. *In vivo*, conventional DC subsets (cDC1and cDC2) and monocyte-derived DC have a different ontogeny (66) and functions (2, 67, 68). Whether these DC subsets share the capacity to form podosomes and migrate in 3D in a protease-dependent manner remains to be explored.

In conclusion, DC adapt their migration mode to the matrix architecture: in a matrix with large pores, they use the amoeboid mode; in a matrix with a low porosity, they use the mesenchymal mode. Interestingly, we demonstrate that mesenchymal migration relies on the capacity to form podosomes and not on the DC maturation status. It is likely that differential regulation of podosome maintenance or dissolution during DC activation has consequences on DC migration in tissues and immune responses during infections and cancer.

## Ethics Statement

For this report, written informed consents were obtained from all the donors under EFS contract n°21/PLER/TOU/IPBS01/2013-0042. According to articles L1243-4 and R1243-61 of the French Public Health Code, the contract was approved by the French Ministry of Science and Technology (agreement number AC 2009-921). All subjects gave written informed consent in accordance with the Declaration of Helsinki.

## Author Contributions

CC and IMP designed the study; EM, GLV, RP, and ON provided reagents and expertize; CC, CL, RG, RM, GLV, and RP performed experiments; CC and IMP wrote the manuscript, all authors provided a final approval of the version to be published.

## Conflict of Interest Statement

The authors declare that the research was conducted in the absence of any commercial or financial relationships that could be construed as a potential conflict of interest.

## Supplementary Material

Figure S1 **(A)** iDC were left untreated or treated with PImix or Y27632 for 16 h, and stained with phalloidin Texas-Red to detect F-actin (red) to reveal podosomes and DAPI to stain nuclei (blue). **(B)** The percentage of cells forming podosomes was quantified. **p* < 0.05. **(C)** iDC seeded on gelatin-FITC-coated glass coverslips were left untreated or treated with PImix or Y27632 for 16 h. Cells were stained with phalloidin Texas-Red to detect F-actin (red) and DAPI to stain nuclei (blue). Dark areas correspond to gelatin-FITC degradation. **(D)** The percentage gelatin-FITC matrix degradation was quantified. Results are expressed as mean ± SEM of at least three independent experiments. ****p* < 0.001 (related to Figure 1).

Figure S2 **(A)** Macrophages and iDC were stained with an anti-vinculin Ab (green), phalloidin Texas-Red to detect F-actin (red) revealing podosomes, and DAPI to stain nuclei (blue). **(B)** The percentage of migrating cells and the mean migration distance of macrophages and iDC migrating in fibrillar collagen I were measured. Results are expressed as mean ± SEM of three independent experiments. **(C)** The percentage of migrating cells and the mean migration distance of macrophages and iDC in Matrigel were measured. Results are expressed as mean ± SEM of three independent experiments. ***p* < 0.01; ****p* < 0.001 compared to iDC condition (related to Figure 1).

Figure S3 **(A)** Histograms showing the MFI of cell-surface maturation markers in iDC and mDC-Pam_3_CSK_4_. Results are expressed as mean ± SEM of 10 independent experiments. **p* < 0.05; ***p* < 0.01; ****p* < 0.001 compared to iDC condition. **(B)** Histograms showing the MFI of cell-surface maturation markers in iDC, mDC-LPS, and iDC+PGE_2_. Results are expressed as mean ± SEM of 10 independent experiments. ***p* < 0.01; ****p* < 0.001 compared to iDC condition (related to Figure 2).

Figure S4 **(A)** Histograms showing the MFI of cell-surface CCR5 or CCR7 in iDC and mDC-LPS. Results are expressed as mean ± SEM of 10 independent experiments. ***p* < 0.01; ****p* < 0.001 compared to iDC condition. **(B)** The percentage of iDC migrating in gelled collagen I was monitored after 24 h when none (white) or CCL5 (grey) was added in the lower chamber as chemoattractant. Results are expressed as mean + SEM of three independent experiments. ***p* < 0.01 compared to none condition. **(C)** quantification of gelatin-FITC degradation by iDC and mDC-LPS left untreated or treated with either CCL5 or CCL19 and CCL21 for 16 h. **(D)** iDC and mDC-LPS seeded on gelatin-FITC-coated glass coverslips were left untreated or stimulated with either CCL5 or CCL19 and CCL21 for 16 h. Cells were stained with phalloidin Texas-Red to detect F-actin (red) and DAPI to stain nuclei (blue). Dark areas correspond to gelatin-FITC degradation. **(E)** The percentage of iDC migrating in fibrillar collagen I was measured when none (white) or CCL5 (grey) was added in the lower chamber as chemoattractant, in control or drug-treated cells (PImix or Y27632). Results are expressed as mean ± SEM of at least three independent experiments. **p* < 0.05 compared to DMSO condition. **(F)** The percentage of mDC-LPS migrating in fibrillar collagen I was measured when none (white) or CCL19 and CCL21 (grey) were added in the lower chamber as chemoattractant, in control or drug-treated cells (PImix or Y27632). Results are expressed as mean ± SEM of at least three independent experiments. **p* < 0.05 compared to DMSO condition. **(G)** The percentage of iDC migrating in Matrigel was measured when none (white) or CCL5 (grey) was added in the lower chamber as chemoattractant, in control or drug-treated cells (PImix or Y27632). Results are expressed as mean ± SEM of at least three independent experiments. **p* < 0.05; ***p* < 0.01 compared to DMSO condition. **(H)** The percentage of iDC migrating in gelled collagen I was measured when none (white) or CCL5 (grey) was added in the lower chamber as chemoattractant, in control or drug-treated cells (PImix or Y27632). Results are expressed as mean ± SEM of at least three independent experiments (related to Figure 3).

Video S1 Immature dendritic cell migrating in 3D matrices. iDC were seeded in transwells filled with fibrillar collagen I, gelled collagen I or Matrigel. z-series of images were acquired after 24 h of migration at the surface of the matrices and at several depth (until 660 μm, 30 μm intervals) into the matrix.

Video S2 Immature dendritic cells migrating in Matrigel. Time-lapse every 10 min during 13.5 h, using the 10× objective of an inverted video microscope.

Video S3 mDC-Pam_3_CSK_4_ migrating in 3D matrices. mDC-Pam_3_CSK_4_ were seeded in transwells filled with fibrillar collagen I, gelled collagen I or Matrigel. z-series of images were acquired after 24 h of migration at the surface of the matrices and at several depth (until 420 μm, 30 μm intervals) into the matrices.

## References

[1] Lammermann T Germain RN. The multiple faces of leukocyte interstitial migration. Semin Immunopathol (2014) 36:227–51. 10.1007/s00281-014-0418-8 24573488 PMC4118216

[2] Worbs T Hammerschmidt SI Forster R. Dendritic cell migration in health and disease. Nat Rev Immunol (2017) 17:30–48. 10.1038/nri.2016.116 27890914

[3] Ng LG Hsu A Mandell MA Roediger B Hoeller C Mrass P Migratory dermal dendritic cells act as rapid sensors of protozoan parasites. PLoS Pathog (2008) 4:e1000222. 10.1371/journal.ppat.1000222 19043558 PMC2583051

[4] Farache J Koren I Milo I Gurevich I Kim KW Zigmond E Luminal bacteria recruit CD103+ dendritic cells into the intestinal epithelium to sample bacterial antigens for presentation. Immunity (2013) 38:581–95. 10.1016/j.immuni.2013.01.009 23395676 PMC4115273

[5] Faure-Andre G Vargas P Yuseff MI Heuze M Diaz J Lankar D Regulation of dendritic cell migration by CD74, the MHC class II-associated invariant chain. Science (2008) 322:1705–10. 10.1126/science.1159894 19074353

[6] Chabaud M Heuze ML Bretou M Vargas P Maiuri P Solanes P Cell migration and antigen capture are antagonistic processes coupled by myosin II in dendritic cells. Nat Commun (2015) 6:7526. 10.1038/ncomms8526 26109323 PMC4491822

[7] Vargas P Chabaud M Thiam HR Lankar D Piel M Lennon-Dumenil AM. Study of dendritic cell migration using micro-fabrication. J Immunol Methods (2016) 432:30–4. 10.1016/j.jim.2015.12.005 26684937

[8] Sallusto F Schaerli P Loetscher P Schaniel C Lenig D Mackay CR Rapid and coordinated switch in chemokine receptor expression during dendritic cell maturation. Eur J Immunol (1998) 28:2760–9. 10.1002/(SICI)1521-4141(199809)28:09<2760::AID-IMMU2760>3.0.CO;2-N 9754563

[9] Sozzani S Allavena P D’amico G Luini W Bianchi G Kataura M Differential regulation of chemokine receptors during dendritic cell maturation: a model for their trafficking properties. J Immunol (1998) 161:1083–6. 9686565

[10] Randolph GJ Angeli V Swartz MA. Dendritic-cell trafficking to lymph nodes through lymphatic vessels. Nat Rev Immunol (2005) 5:617–28. 10.1038/nri1670 16056255

[11] Schumann K Lammermann T Bruckner M Legler DF Polleux J Spatz JP Immobilized chemokine fields and soluble chemokine gradients cooperatively shape migration patterns of dendritic cells. Immunity (2010) 32:703–13. 10.1016/j.immuni.2010.04.017 20471289

[12] Platt AM Randolph GJ. Dendritic cell migration through the lymphatic vasculature to lymph nodes. Adv Immunol (2013) 120:51–68. 10.1016/B978-0-12-417028-5.00002-8 24070380

[13] Weber M Hauschild R Schwarz J Moussion C De Vries I Legler DF Interstitial dendritic cell guidance by haptotactic chemokine gradients. Science (2013) 339:328–32. 10.1126/science.1228456 23329049

[14] Wolf K Muller R Borgmann S Brocker EB Friedl P. Amoeboid shape change and contact guidance: T-lymphocyte crawling through fibrillar collagen is independent of matrix remodeling by MMPs and other proteases. Blood (2003) 102:3262–9. 10.1182/blood-2002-12-3791 12855577

[15] Lämmermann T Bader B Monkley S Worbs T Wedlich-Söldner R Hirsch K Rapid leukocyte migration by integrin-independent flowing and squeezing. Nature (2008) 453:51–5. 10.1038/nature06887 18451854

[16] Lammermann T Renkawitz J Wu X Hirsch K Brakebusch C Sixt M. Cdc42-dependent leading edge coordination is essential for interstitial dendritic cell migration. Blood (2009) 113:5703–10. 10.1182/blood-2008-11-191882 19190242

[17] Lammermann T Sixt M. Mechanical modes of ‘amoeboid’ cell migration. Curr Opin Cell Biol (2009) 21:636–44. 10.1016/j.ceb.2009.05.003 19523798

[18] Sabeh F Shimizu-Hirota R Weiss SJ. Protease-dependent versus -independent cancer cell invasion programs: three-dimensional amoeboid movement revisited. J Cell Biol (2009) 185:11–9. 10.1083/jcb.200807195 19332889 PMC2700505

[19] Vigl B Aebischer D Nitschke M Iolyeva M Rothlin T Antsiferova O Tissue inflammation modulates gene expression of lymphatic endothelial cells and dendritic cell migration in a stimulus-dependent manner. Blood (2011) 118:205–15. 10.1182/blood-2010-12-326447 21596851

[20] Nitschke M Aebischer D Abadier M Haener S Lucic M Vigl B Differential requirement for ROCK in dendritic cell migration within lymphatic capillaries in steady-state and inflammation. Blood (2012) 120:2249–58. 10.1182/blood-2012-03-417923 22855606

[21] Vargas P Barbier L Saez PJ Piel M. Mechanisms for fast cell migration in complex environments. Curr Opin Cell Biol (2017) 48:72–8. 10.1016/j.ceb.2017.04.007 28641118

[22] Linder S Wiesner C. Tools of the trade: podosomes as multipurpose organelles of monocytic cells. Cell Mol Life Sci (2015) 72:121–35. 10.1007/s00018-014-1731-z 25300510 PMC11113205

[23] Calle Y Burns S Thrasher AJ Jones GE The leukocyte podosome. Eur J Cell Biol (2006) 85:151–7. 10.1016/j.ejcb.2005.09.003 16546557

[24] Labernadie A Thibault C Vieu C Maridonneau-Parini I Charrière G. Dynamics of podosome stiffness revealed by atomic force microscopy. Proc Natl Acad Sci U S A (2010) 107:21016–21. 10.1073/pnas.1007835107 21081699 PMC3000246

[25] Labernadie A Bouissou A Delobelle P Balor S Voituriez R Proag A Protrusion force microscopy reveals oscillatory force generation and mechanosensing activity of human macrophage podosomes. Nat Commun (2014) 5:5343. 10.1038/ncomms6343 25385672

[26] Maridonneau-Parini I. Control of macrophage 3D migration: a therapeutic challenge to limit tissue infiltration. Immunol Rev (2014) 262:216–31. 10.1111/imr.12214 25319337

[27] Wiesner C Le-Cabec V El Azzouzi K Maridonneau-Parini I Linder S. Podosomes in space: macrophage migration and matrix degradation in 2D and 3D settings. Cell Adh Migr (2014) 8:179–91. 10.4161/cam.28116 24713854 PMC4198342

[28] Bouissou A Proag A Bourg N Pingris K Cabriel C Balor S Podosome force generation machinery: a local balance between protrusion at the core and traction at the ring. ACS Nano (2017) 11:4028–40. 10.1021/acsnano.7b00622 28355484

[29] Van Goethem E Poincloux R Gauffre F Maridonneau-Parini I Le Cabec V. Matrix architecture dictates three-dimensional migration modes of human macrophages: differential involvement of proteases and podosome-like structures. J Immunol (2010) 184:1049–61. 10.4049/jimmunol.0902223 20018633

[30] Van Goethem E Guiet R Balor S Charriere GM Poincloux R Labrousse A Macrophage podosomes go 3D. Eur J Cell Biol (2011) 90:224–36. 10.1016/j.ejcb.2010.07.011 20801545

[31] Verollet C Gallois A Dacquin R Lastrucci C Pandruvada SN Ortega N Hck contributes to bone homeostasis by controlling the recruitment of osteoclast precursors. FASEB J (2013) 27:3608–18. 10.1096/fj.13-232736 23742809 PMC4046168

[32] Burns S Thrasher AJ Blundell MP Machesky L Jones GE. Configuration of human dendritic cell cytoskeleton by Rho GTPases, the WAS protein, and differentiation. Blood (2001) 98:1142–9. 10.1182/blood.V98.4.1142 11493463

[33] West MA Wallin RP Matthews SP Svensson HG Zaru R Ljunggren HG Enhanced dendritic cell antigen capture via toll-like receptor-induced actin remodeling. Science (2004) 305:1153–7. 10.1126/science.1099153 15326355

[34] van Helden SF Van Den Dries K Oud MM Raymakers RA Netea MG Van Leeuwen FN TLR4-mediated podosome loss discriminates gram-negative from gram-positive bacteria in their capacity to induce dendritic cell migration and maturation. J Immunol (2010) 184:1280–91. 10.4049/jimmunol.0900764 20038642

[35] Troegeler A Lastrucci C Duval C Tanne A Cougoule C Maridonneau-Parini I An efficient siRNA-mediated gene silencing in primary human monocytes, dendritic cells and macrophages. Immunol Cell Biol (2014) 92:699–708. 10.1038/icb.2014.39 24890643

[36] Guiet R Van Goethem E Cougoule C Balor S Valette A Al Saati T The process of macrophage migration promotes matrix metalloproteinase-independent invasion by tumor cells. J Immunol (2011) 187:3806–14. 10.4049/jimmunol.1101245 21880978 PMC4276309

[37] Verollet C Souriant S Bonnaud E Jolicoeur P Raynaud-Messina B Kinnaer C HIV-1 reprograms the migration of macrophages. Blood (2015) 125:1611–22. 10.1182/blood-2014-08-596775 25527710

[38] Cougoule C Van Goethem E Le Cabec V Lafouresse F Dupre L Mehraj V Blood leukocytes and macrophages of various phenotypes have distinct abilities to form podosomes and to migrate in 3D environments. Eur J Cell Biol (2012) 91:938–49. 10.1016/j.ejcb.2012.07.002 22999511

[39] Cougoule C Le Cabec V Poincloux R Al Saati T Mege JL Tabouret G Three-dimensional migration of macrophages requires Hck for podosome organization and extracellular matrix proteolysis. Blood (2010) 115:1444–52. 10.1182/blood-2009-04-218735 19897576 PMC5070714

[40] Lastrucci C Benard A Balboa L Pingris K Souriant S Poincloux R Tuberculosis is associated with expansion of a motile, permissive and immunomodulatory CD16(+) monocyte population via the IL-10/STAT3 axis. Cell Res (2015) 25:1333–51. 10.1038/cr.2015.123 26482950 PMC4670988

[41] LeBleu VS Macdonald B Kalluri R Structure and function of basement membranes. Exp Biol Med (Maywood) (2007) 232:1121–9. 10.3181/0703-MR-72 17895520

[42] Jevnikar Z Mirkovic B Fonovic UP Zidar N Svajger U Kos J. Three-dimensional invasion of macrophages is mediated by cysteine cathepsins in protrusive podosomes. Eur J Immunol (2012) 42:3429–41. 10.1002/eji.201242610 23018451

[43] Calle Y Carragher NO Thrasher AJ Jones GE Inhibition of calpain stabilises podosomes and impairs dendritic cell motility. J Cell Sci (2006) 119:2375–85. 10.1242/jcs.02939 16723743

[44] van Helden SF Krooshoop DJ Broers KC Raymakers RA Figdor CG Van Leeuwen FN. A critical role for prostaglandin E2 in podosome dissolution and induction of high-speed migration during dendritic cell maturation. J Immunol (2006) 177:1567–74. 10.4049/jimmunol.177.3.1567 16849464

[45] Nourshargh S Hordijk PL Sixt M. Breaching multiple barriers: leukocyte motility through venular walls and the interstitium. Nat Rev Mol Cell Biol (2010) 11:366–78. 10.1038/nrm2889 20414258

[46] van Helden SF Oud MM Joosten B Peterse N Figdor CG Van Leeuwen FN. PGE2-mediated podosome loss in dendritic cells is dependent on actomyo-sin contraction downstream of the RhoA-Rho-kinase axis. J Cell Sci (2008) 121:1096–106. 10.1242/jcs.020289 18334555

[47] Salvi V Vaira X Gianello V Vermi W Bugatti M Sozzani S TLR signalling pathways diverge in their ability to induce PGE2. Mediators Inflamm (2016) 2016:5678046. 10.1155/2016/5678046 27630451 PMC5007370

[48] Gui P Labrousse A Van Goethem E Besson A Maridonneau-Parini I Le Cabec V. Rho/ROCK pathway inhibition by the CDK inhibitor p27(kip1) participates in the onset of macrophage 3D-mesenchymal migration. J Cell Sci (2014) 127:4009–23. 10.1242/jcs.150987 25015295

[49] Guiet R Verollet C Lamsoul I Cougoule C Poincloux R Labrousse A Macrophage mesenchymal migration requires podosome stabiliza-tion by filamin A. J Biol Chem (2012) 287:13051–62. 10.1074/jbc.M111.307124 22334688 PMC3339984

[50] Park H Dovas A Hanna S Lastrucci C Cougoule C Guiet R Tyrosine phosphorylation of Wiskott-Aldrich syndrome protein (WASP) by Hck regulates macrophage function. J Biol Chem (2014) 289:7897–906. 10.1074/jbc.M113.509497 24482227 PMC3953300

[51] Weidner JM Kanatani S Hernandez-Castaneda MA Fuks JM Rethi B Wallin RP Rapid cytoskeleton remodelling in dendritic cells following invasion by *Toxoplasma gondii* coincides with the onset of a hypermigratory phenotype. Cell Microbiol (2013) 15:1735–52. 10.1111/cmi.12145 23534541

[52] Olafsson EB Varas-Godoy M Barragan A. *Toxoplasma gondii* infection shifts dendritic cells into an amoeboid rapid migration mode encompassing podosome dissolution, secretion of TIMP-1, and reduced proteolysis of extracellular matrix. Cell Microbiol (2018) 20(3). 10.1111/cmi.12808 29119662

[53] Kanatani S Uhlen P Barragan A. Infection by *Toxoplasma gondii* induces amoeboid-like migration of dendritic cells in a three-dimensional collagen matrix. PLoS One (2015) 10:e0139104. 10.1371/journal.pone.0139104 26406763 PMC4583262

[54] Le Roux-Goglin E Varon C Spuul P Asencio C Megraud F Genot E. Helicobacter infection induces podosome assembly in primary hepatocytes in vitro. Eur J Cell Biol (2012) 91:161–70. 10.1016/j.ejcb.2011.11.003 22306377

[55] Scandella E Men Y Legler DF Gillessen S Prikler L Ludewig B CCL19/CCL21-triggered signal transduction and migration of dendritic cells requires prostaglandin E2. Blood (2004) 103:1595–601. 10.1182/blood-2003-05-1643 14592837

[56] Legler DF Krause P Scandella E Singer E Groettrup M. Prostaglandin E2 is generally required for human dendritic cell migration and exerts its effect via EP2 and EP4 receptors. J Immunol (2006) 176:966–73. 10.4049/jimmunol.176.2.966 16393982

[57] Yen JH Kocieda VP Jing H Ganea D. Prostaglandin E2 induces matrix metalloproteinase 9 expression in dendritic cells through two independent signaling pathways leading to activator protein 1 (AP-1) activation. J Biol Chem (2011) 286:38913–23. 10.1074/jbc.M111.252932 21940623 PMC3234716

[58] Chabot V Reverdiau P Iochmann S Rico A Senecal D Goupille C CCL5-enhanced human immature dendritic cell migration through the basement membrane in vitro depends on matrix metalloproteinase-9. J Leukoc Biol (2006) 79:767–78. 10.1189/jlb.0804464 16434695

[59] Yang MX Qu X Kong BH Lam QL Shao QQ Deng BP Membrane type 1-matrix metalloproteinase is involved in the migration of human monocyte-derived dendritic cells. Immunol Cell Biol (2006) 84:557–62. 10.1111/j.1440-1711.2006.01465.x 16956391

[60] West MA Prescott AR Chan KM Zhou Z Rose-John S Scheller J TLR ligand-induced podosome disassembly in dendritic cells is ADAM17 dependent. J Cell Biol (2008) 182:993–1005. 10.1083/jcb.200801022 18762577 PMC2528573

[61] Gawden-Bone C Zhou Z King E Prescott A Watts C Lucocq J. Dendritic cell podosomes are protrusive and invade the extracellular matrix using metalloproteinase MMP-14. J Cell Sci (2010) 123:1427–37. 10.1242/jcs.056515 20356925 PMC2858019

[62] Wiesner C Faix J Himmel M Bentzien F Linder S. KIF5B and KIF3A/KIF3B kinesins drive MT1-MMP surface exposure, CD44 shedding, and extracellular matrix degradation in primary macrophages. Blood (2010) 116:1559–69. 10.1182/blood-2009-12-257089 20505159

[63] Baranov M Ter Beest M Reinieren-Beeren I Cambi A Figdor CG Van Den Bogaart G. Podosomes of dendritic cells facilitate antigen sampling. J Cell Sci (2014) 127:1052–64. 10.1242/jcs.141226 24424029 PMC4054684

[64] Ratzinger G Stoitzner P Ebner S Lutz MB Layton GT Rainer C Matrix metalloproteinases 9 and 2 are necessary for the migration of Langerhans cells and dermal dendritic cells from human and murine skin. J Immunol (2002) 168:4361–71. 10.4049/jimmunol.168.9.4361 11970978

[65] Zelenay S Van Der Veen AG Bottcher JP Snelgrove KJ Rogers N Acton SE Cyclooxygenase-dependent tumor growth through evasion of immunity. Cell (2015) 162:1257–70. 10.1016/j.cell.2015.08.015 26343581 PMC4597191

[66] Guilliams M Ginhoux F Jakubzick C Naik SH Onai N Schraml BU Dendritic cells, monocytes and macrophages: a unified nomenclature based on ontogeny. Nat Rev Immunol (2014) 14:571–8. 10.1038/nri3712 25033907 PMC4638219

[67] Hansen M Andersen MH The role of dendritic cells in cancer. Semin Immunopathol (2017) 39:307–16. 10.1007/s00281-016-0592-y 27638181

[68] Keirsse J Van Damme H Van Ginderachter JA Laoui D. Exploiting tumor-associated dendritic cell heterogeneity for novel cancer therapies. J Leukoc Biol (2017) 102:317–24. 10.1189/jlb.4MR1116-466R 28389620

