# Supplementary figures and images for: Podosomes, But Not the Maturation Status, Determine the Protease-Dependent 3D Migration in Human Dendritic Cells
Source: Front Immunol. 2018 Apr 30;9:846. doi: 10.3389/fimmu.2018.00846 (PMC5936769; doi:10.3389/fimmu.2018.00846)

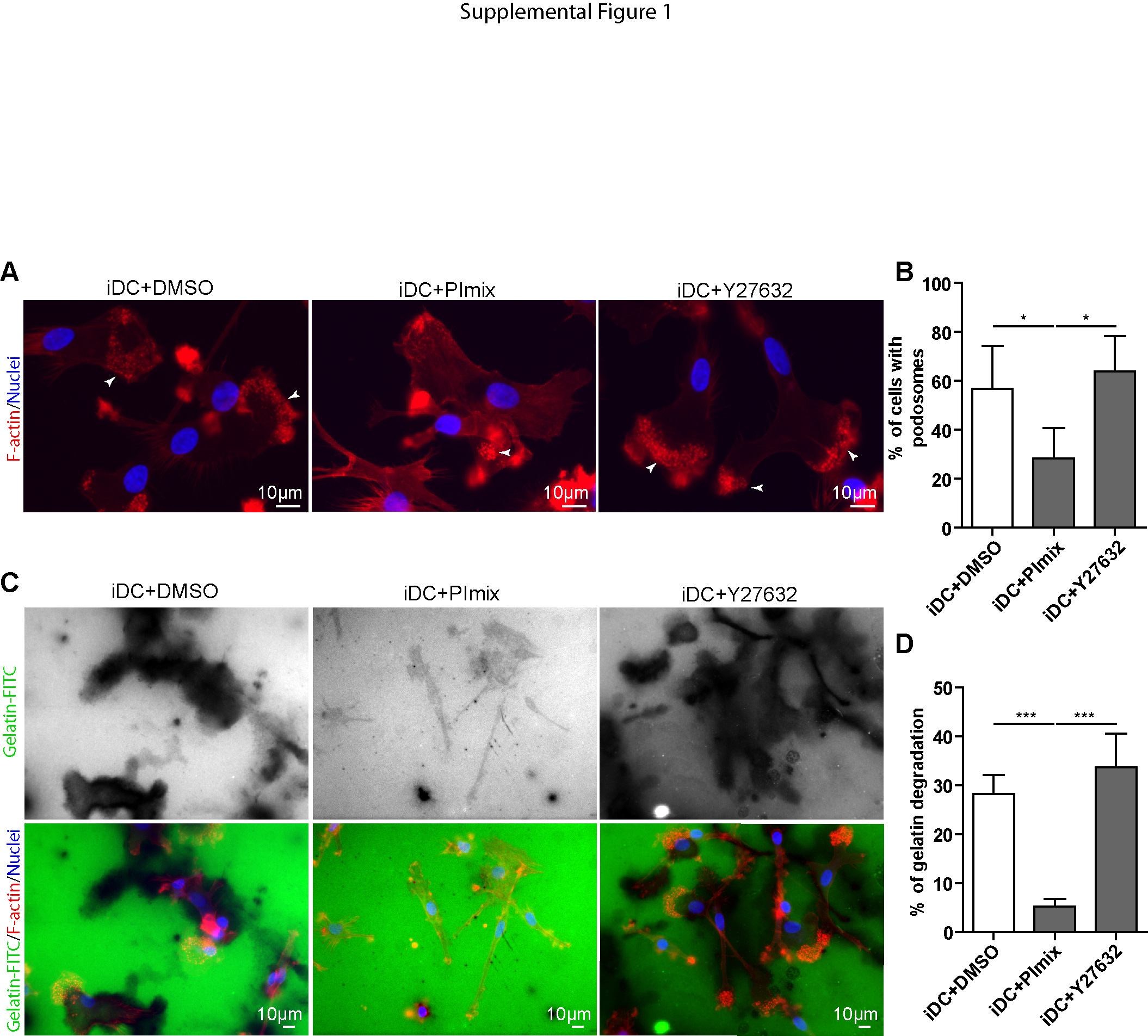

Supplement: Figure S1 — (A) iDC were left untreated or treated with PImix or Y27632 for 16 h, and stained with phalloidin Texas-Red to detect F-actin (red) to reveal podosomes and DAPI to stain nuclei (blue). (B) The percentage of cells forming podosomes was quantified. *p < 0.05. (C) iDC seeded on gelatin-FITC-coated glass coverslips were left untreated or treated with PImix or Y27632 for 16 h. Cells were stained with phalloidin Texas-Red to detect F-actin (red) and DAPI to stain nuclei (blue). Dark areas correspond to gelatin-FITC degradation. (D) The percentage gelatin-FITC matrix degradation was quantified. Results are expressed as mean ± SEM of at least three independent experiments. ***p < 0.001 (related to Figure 1). [file Image_1.jpeg]

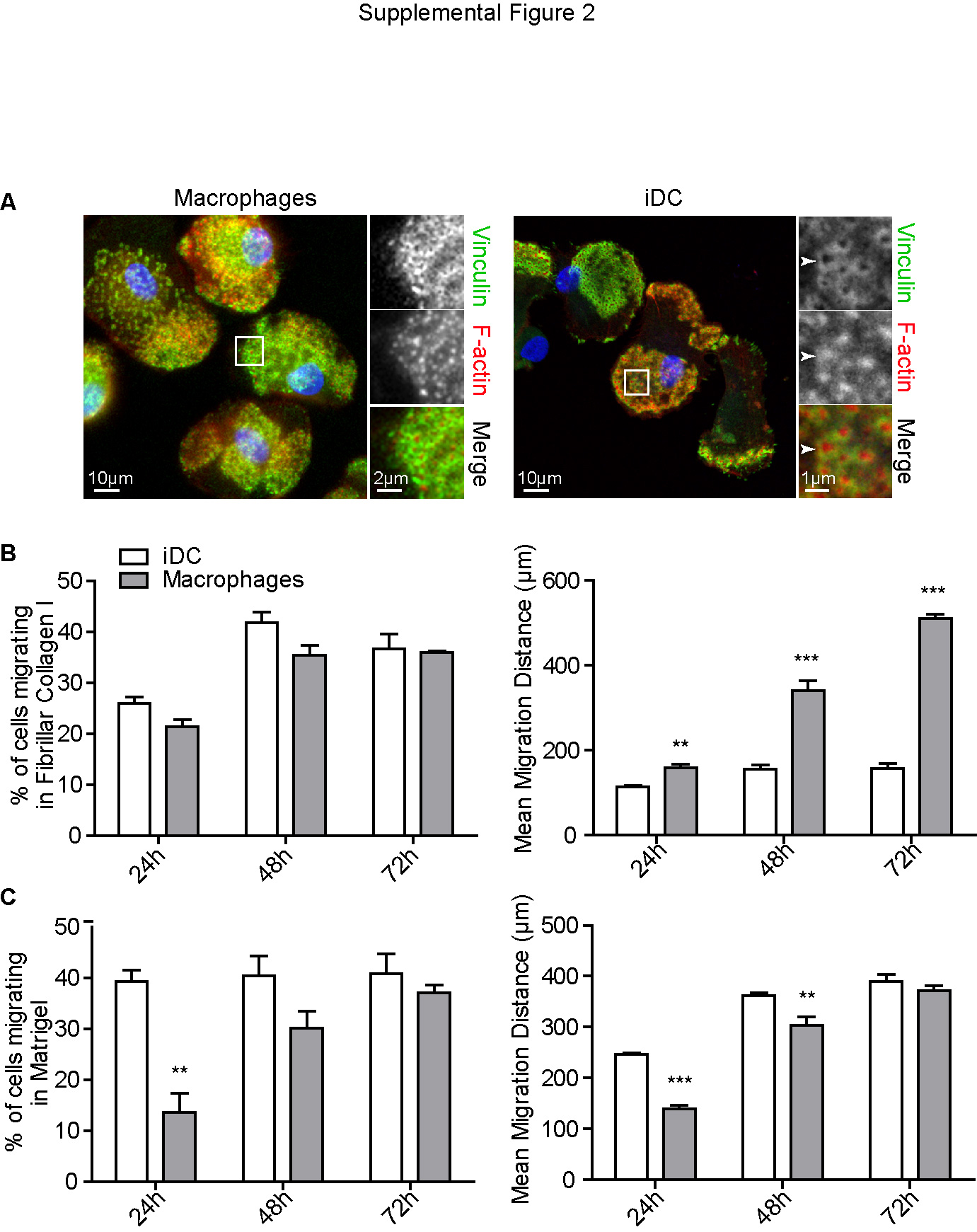

Supplement: Figure S2 — (A) Macrophages and iDC were stained with an anti-vinculin Ab (green), phalloidin Texas-Red to detect F-actin (red) revealing podosomes, and DAPI to stain nuclei (blue). (B) The percentage of migrating cells and the mean migration distance of macrophages and iDC migrating in fibrillar collagen I were measured. Results are expressed as mean ± SEM of three independent experiments. (C) The percentage of migrating cells and the mean migration distance of macrophages and iDC in Matrigel were measured. Results are expressed as mean ± SEM of three independent experiments. **p < 0.01; ***p < 0.001 compared to iDC condition (related to Figure 1). [file Image_2.jpeg]

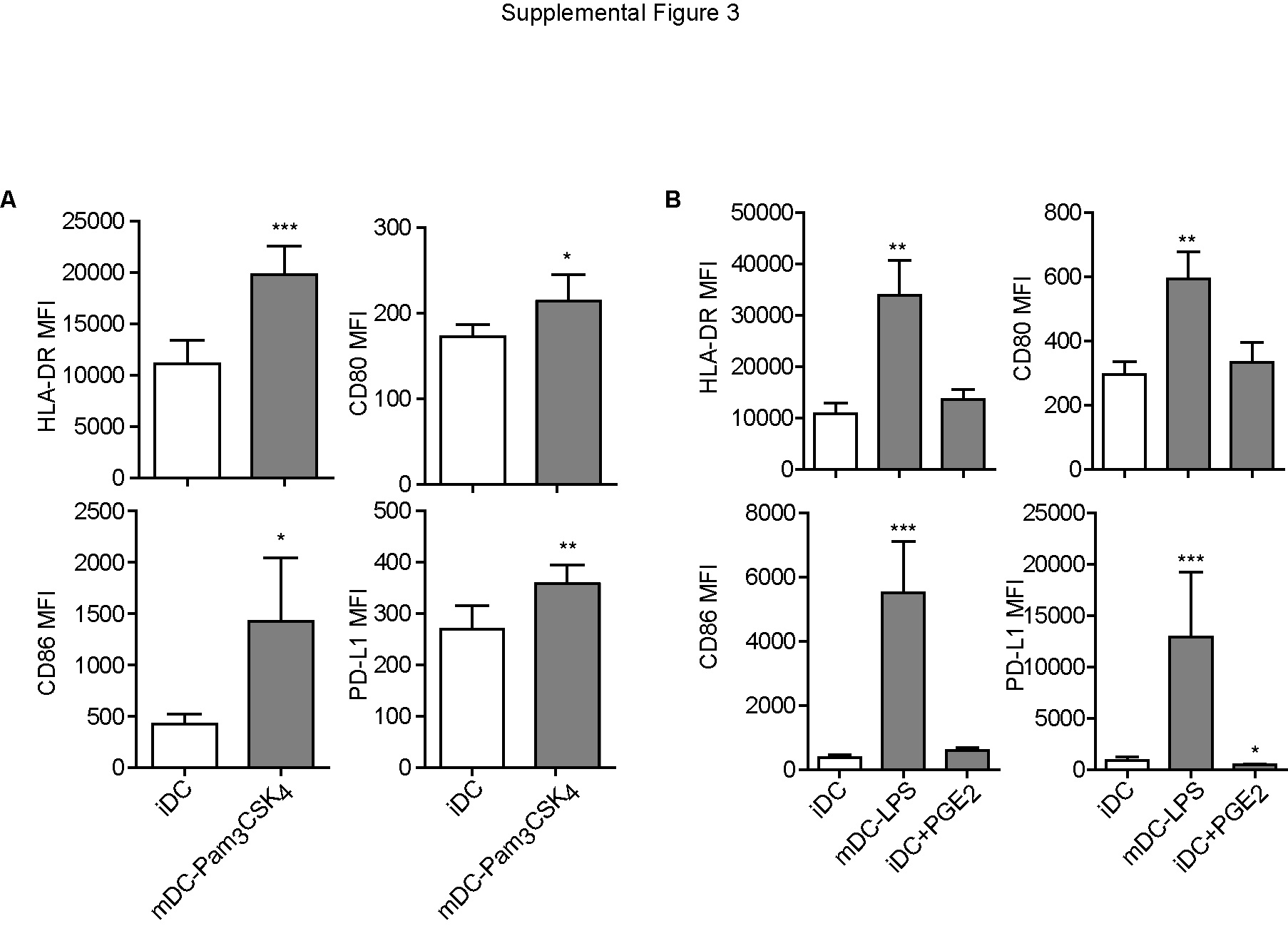

Supplement: Figure S3 — (A) Histograms showing the MFI of cell-surface maturation markers in iDC and mDC-Pam3CSK4. Results are expressed as mean ± SEM of 10 independent experiments. *p < 0.05; **p < 0.01; ***p < 0.001 compared to iDC condition. (B) Histograms showing the MFI of cell-surface maturation markers in iDC, mDC-LPS, and iDC+PGE2. Results are expressed as mean ± SEM of 10 independent experiments. **p < 0.01; ***p < 0.001 compared to iDC condition (related to Figure 2). [file Image_3.jpeg]

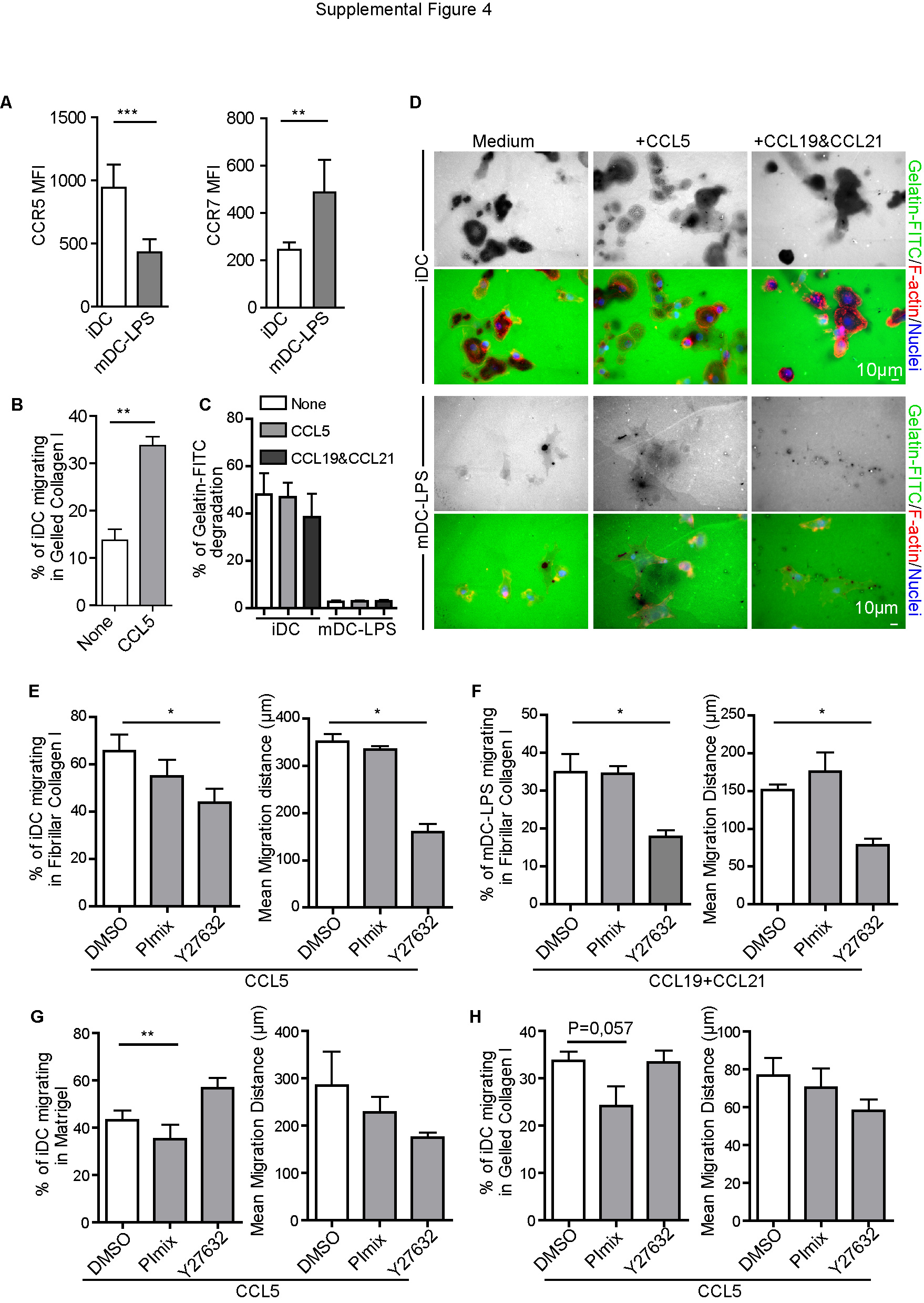

Supplement: Figure S4 — (A) Histograms showing the MFI of cell-surface CCR5 or CCR7 in iDC and mDC-LPS. Results are expressed as mean ± SEM of 10 independent experiments. **p < 0.01; ***p < 0.001 compared to iDC condition. (B) The percentage of iDC migrating in gelled collagen I was monitored after 24 h when none (white) or CCL5 (grey) was added in the lower chamber as chemoattractant. Results are expressed as mean + SEM of three independent experiments. **p < 0.01 compared to none condition. (C) quantification of gelatin-FITC degradation by iDC and mDC-LPS left untreated or treated with either CCL5 or CCL19 and CCL21 for 16 h. (D) iDC and mDC-LPS seeded on gelatin-FITC-coated glass coverslips were left untreated or stimulated with either CCL5 or CCL19 and CCL21 for 16 h. Cells were stained with phalloidin Texas-Red to detect F-actin (red) and DAPI to stain nuclei (blue). Dark areas correspond to gelatin-FITC degradation. (E) The percentage of iDC migrating in fibrillar collagen I was measured when none (white) or CCL5 (grey) was added in the lower chamber as chemoattractant, in control or drug-treated cells (PImix or Y27632). Results are expressed as mean ± SEM of at least three independent experiments. *p < 0.05 compared to DMSO condition. (F) The percentage of mDC-LPS migrating in fibrillar collagen I was measured when none (white) or CCL19 and CCL21 (grey) were added in the lower chamber as chemoattractant, in control or drug-treated cells (PImix or Y27632). Results are expressed as mean ± SEM of at least three independent experiments. *p < 0.05 compared to DMSO condition. (G) The percentage of iDC migrating in Matrigel was measured when none (white) or CCL5 (grey) was added in the lower chamber as chemoattractant, in control or drug-treated cells (PImix or Y27632). Results are expressed as mean ± SEM of at least three independent experiments. *p < 0.05; **p < 0.01 compared to DMSO condition. (H) The percentage of iDC migrating in gelled collagen I was measured when none (white) or CCL [file Image_4.jpeg]
