# Supplementary material for: Podosomes, But Not the Maturation Status, Determine the Protease-Dependent 3D Migration in Human Dendritic Cells
Source: Front Immunol. 2018 Apr 30;9:846. doi: 10.3389/fimmu.2018.00846 (PMC5936769; doi:10.3389/fimmu.2018.00846)
Supplement: Supplementary file 8 [file Presentation_1.PDF]

Podosomes, but not the maturation status, determine the protease-dependent 3D migration in human dendritic cells

Céline Cougoule[1*], Claire Lastrucci[1], Romain Guiet[1], Rémi Mascarau[1], Etienne Meunier[1], Geanncarlo Lugo-Villarino[1], Olivier Neyrolles[1], Renaud Poincloux[1] and Isabelle Maridonneau-Parini[1*]

Supplemental Figure Legend

Supplemental Figure 1 (related to Figure 1): **A**, iDC were left untreated or treated with PImix or Y27632 for 16 h, and stained with phalloidin Texas-Red to detect F-actin (red) to reveal podosomes and DAPI to stain nuclei (blue). **B**, The percentage of cells forming podosomes was quantified. *, P<0,05. **C**, iDC seeded on gelatin-FITC-coated glass coverslips were left untreated or treated with PImix or Y27632 for 16 h. Cells were stained with phalloidin Texas-Red to detect F-actin (red) and DAPI to stain nuclei (blue). Dark areas correspond to gelatin-FITC degradation. **D**, The percentage gelatin-FITC matrix degradation was quantified. Results are expressed as mean ± SEM of at least three independent experiments. ***, P<0.001.

Supplemental Figure 2 (related to Figure 1): A, Macrophages and iDC were stained with an anti-vinculin Ab (green), phalloidin Texas-Red to detect F-actin (red) revealing podosomes and DAPI to stain nuclei (blue). **B**, The percentage of migrating cells and the mean migration distance of macrophages and iDC migrating in fibrillar collagen I were measured. Results are expressed as mean ± SEM of three independent experiments. **C**, The percentage of migrating cells and the mean migration distance of macrophages and iDC in Matrigel were measured. Results are expressed as mean ± SEM of three independent experiments. **, P<0,01; ***, P<0.001 compared to iDC condition.

Supplemental Figure 3 (related to Figure 2): A, Histograms showing the MFI of cell-surface maturation markers in iDC and mDC-Pam_3CSK_4. Results are expressed as mean ± SEM of ten independent experiments. *, P<0,05; **, P<0,01; ***, P<0.001 compared to iDC condition. **B**, Histograms showing the MFI of cell-surface maturation markers in iDC, mDC-LPS and iDC+PGE_2. Results are expressed as mean ± SEM of ten independent experiments. **, P<0,01; ***, P<0.001 compared to iDC condition.

Supplemental Figure 4 (related to Figure 3): **A**, Histograms showing the MFI of cell-surface CCR5 or CCR7 in iDC and mDC-LPS. Results are expressed as mean ± SEM of ten independent experiments. **, P<0,01; ***, P<0.001 compared to iDC condition. **B**, The percentage of iDC migrating in gelled collagen I was monitored after 24 h when none (white) or CCL5 (grey) was added in the lower chamber as chemoattractant. Results are expressed as mean + SEM of three independent experiments. **, P<0,01 compared to none condition. **C**, quantification of gelatin-FITC degradation by iDC and mDC-LPS left untreated or treated with either CCL5 or CCL19 and CCL21 for 16 h. **D**, iDC and mDC-LPS seeded on gelatin-FITC-coated glass coverslips were left untreated or stimulated with either CCL5 or CCL19 and CCL21 for 16 h. Cells were stained with phalloidin Texas-Red to detect F-actin (red) and DAPI to stain nuclei (blue). Dark areas correspond to gelatin-FITC degradation. **E**, The percentage of iDC migrating in fibrillar collagen I was measured when none (white) or CCL5 (grey) was added in the lower chamber as chemoattractant, in control or drug-treated cells (PImix or Y27632). Results are expressed as mean ± SEM of at least three independent experiments. *, P<0,05 compared to DMSO condition. **F**, The percentage of mDC-LPS migrating in fibrillar collagen I was measured when none (white) or CCL19 and CCL21 (grey) were added in the lower chamber as chemoattractant, in control or drug-treated cells (PImix or Y27632). Results are expressed as mean ± SEM of at least three independent experiments. *, P<0,05 compared to DMSO condition. **G**, The percentage of iDC migrating in Matrigel was measured when none (white) or CCL5 (grey) was added in the lower chamber as chemoattractant, in control or drug-treated cells (PImix or Y27632). Results are expressed as mean ± SEM of at least three independent experiments. *, P<0,05; **, P<0,01 compared to DMSO condition. **H**, The percentage of iDC migrating in gelled collagen I was measured when none (white) or CCL5 (grey) was added in the lower chamber as chemoattractant, in control or drug-treated cells

(PImix or Y27632). Results are expressed as mean ± SEM of at least three independent experiments.

Video 1: Immature dendritic cell migrating in 3D matrices.

iDCs were seeded in transwells filled with Fibrillar collagen I, gelled collagen I or Matrigel. z-series of images were acquired after 24h of migration at the surface of the matrices and at several depth (until 660µm, 30 µm intervals) into the matrix.

Video 2: Immature dendritic cells migrating in Matrigel. Time-lapse every 10 min during 13.5 h, using the 10× objective of an inverted video microscope.

Video 3: mDC-Pam_3CSK_4 migrating in 3D matrices.

mDC-Pam_3CSK_4 were seeded in transwells filled with Fibrillar collagen I, gelled collagen I or Matrigel. z-series of images were acquired after 24h of migration at the surface of the matrices and at several depth (until 420µm, 30 µm intervals) into the matrix.
